# Setting health systems research priorities for Afghanistan: an application of the child health and nutrition research initiative (CHNRI) methodology to set a roadmap to 2030

**DOI:** 10.1136/bmjgh-2024-018578

**Published:** 2025-07-25

**Authors:** Sama El Baz, Emily C Keats, Hana Tasic, Robert Black, David H Peters, Najibullah Safi, Hannah Tappis, Kerri Wazny, Nadia Akseer, Afghanistan Health Systems Research Prioritization Collaborators, Attaullah Ahmadi

**Affiliations:** 1 Johns Hopkins Bloomberg School of Public Health, Baltimore, Maryland, USA; 2 Modern Scientist Global, St. Catharines, Niagara, Canada; 3 York University, Toronto, Ontario, Canada; 4 Independent consultant, Kabul, Afghanistan; 5 Jhpiego, Baltimore, Maryland, USA

**Keywords:** Global Health, Health systems, Health systems evaluation

## Abstract

**Introduction:**

Afghanistan’s health system has faced considerable challenges since the Taliban takeover in 2021, leaving the population vulnerable to an increased risk of morbidity and mortality. Research to illuminate the current functioning of the health system and approaches for strengthening its key components is critically needed to address imminent and evolving health needs of the Afghan people.

**Methods:**

A health systems’ research agenda for Afghanistan that uses a systematic and evidence-based *wisdom of the crowds’* approach has yet to be developed. Using the Child Health and Nutrition Research Initiative methodology, this study identifies the top 20 health systems’ research priorities among experienced Afghanistan health researchers. Priorities were also considered when disaggregating data by subgroups, such as Afghan versus non-Afghan respondents and those from low- and middle-income versus high-income settings.

**Results:**

A total of 303 researchers were invited to score the research questions; 86 responded to the scoring survey and 55 completed it (60% were of Afghan origin). The highest priority questions were relatively diverse in terms of topic area, with questions spanning system-level factors, healthcare quality, community-based healthcare, improvements in the pharmaceutical sector, epidemiological trends, health management information systems and surveillance, access to care and approaches to improving service delivery in Afghanistan, among many others. ‘Delivery’-focused and ‘development’-focused questions were prioritised, demonstrating that participants assigned greater importance to more practical research questions that would explore features of and approaches to improving existing health system structures within the current Afghan context. Results were consistent across subgroups.

**Conclusion:**

This research prioritisation exercise fills a gap by generating consensus and establishing a research agenda for strengthening Afghanistan’s health system.

**WHAT IS ALREADY KNOWN ON THIS TOPIC**

- To support the resilience of Afghanistan’s health system following the 2021 transition of power, a health systems’ research agenda is required to focus on areas of greatest need to improve impact, access, equity and effectiveness of a resource-constrained health system.

**WHAT THIS STUDY ADDS**

- Using the systematic and evidence-based CHNRI methodology, this study identifies the top 20 health systems’ research priorities among experienced Afghanistan health researchers. Research priorities were diverse in terms of topic area, though half of the top 20 were ‘delivery’ questions, highlighting the importance of optimising health through means that are already available in the Afghan context.

**HOW THIS STUDY MIGHT AFFECT RESEARCH, PRACTICE OR POLICY**

- This study lists the most pressing health research priorities in Afghanistan today. Addressing these research priorities would support Afghanistan’s Health Sector Transition Strategy and, ultimately, inform a more effective implementation of health services and interventions by national and international organisations.

## Introduction

 One of the world’s largest humanitarian crises is currently ensuing in Afghanistan, where 23.7 million out of the 40 million population require urgent humanitarian assistance to survive.^1^ On 15 August 2021, after more than four decades of war, the Islamic Republic of Afghanistan fell to the Taliban following the withdrawal of the United States military forces. After these events, key international donor agencies suspended development assistance, which funded more than 75% of Afghanistan’s public expenditures, including the public health system.^2^

This series of events exacerbated the pre-existing economic crisis and resulted in social collapse, with poverty skyrocketing from 49.4% in 2020 to 85% of the population in 2023.^3^ Compounded by a 3-year drought and other natural disasters, upwards of 14.8 million people, or one in three Afghans, are now projected to face hunger and starvation in 2025 with 3.1 million at emergency levels.^4^ Malnutrition is a significant problem: 875 000 children under five have severe acute malnutrition, while 2.3 million children and 840 000 women suffer from moderate acute malnutrition.^5 6^

The defacto authorities have issued policies banning or greatly restricting women and girls from basic participation in public life, impacting their human rights.^7^ They have forbidden women’s work outside the home, do not allow free movement without male relative accompaniment, secondary and higher education are not permitted and access to healthcare, for both women and their children, is challenging.^1 7^

The events and policies enacted since August 2021 have had a direct impact on the health of all Afghans and threaten to undo progress achieved over the past two decades towards Afghanistan’s Sustainable Development Goals. From 2000 to 2018, but particularly through the 2000s, coverage of health services improved and Afghanistan’s maternal mortality rate decreased nearly 64%, from 1450 deaths per 100 000 live births to 638 deaths.^8 9^ Also during this period, mortality in children under five decreased nearly 29%, from 127.8 deaths per 1000 live births to 91.0 deaths, and neonatal mortality was similarly reduced.^9^ Though the country had been moving towards universal health coverage with the proposed Integrated Package of Essential Health Services, the Taliban takeover meant that this suite of services was never implemented.^10^ As a result, in its current state, the Afghan health system is ill-equipped to address the health and nutrition needs of the population.

Data on epidemiological trends and intervention access and uptake are critically needed to design and implement effective and timely strategies within current restrictions and realities that include those related to funding, travel and women’s societal participation. However, health research activities and studies were largely suspended after the withdrawal of international financial support for much of the health system. This lack of data directly impacts the ability to plan, allocate funding, implement interventions and monitor health services appropriate to the current needs of the population.

Addressing the current health needs of the Afghan population will require careful consideration of the evidence to establish an agenda for the allocation of funding and insight into how services can be effectively delivered within current constraints. Developing a list of research priorities will be critical to guide donor agencies and national and international researchers in their pursuit of Afghanistan health-related research. Currently, the diversity in existing research areas along with the enormity and variety of health needs results in many competing priorities. As such, a systematic method is needed to prioritise health needs and establish acceptable means for delivery of related interventions. This paper reports on Afghanistan’s first health research prioritisation exercise that engages a wide variety of Afghan national and international stakeholders and uses the well-established and tested methodology, the Child Health and Nutrition Research Initiative (CHNRI).

## Methods

The CHNRI method, described in greater detail elsewhere,^11 12^ was used to set an agenda for health research priorities for Afghanistan. This method has been used 100+ times across a broad array of health disciplines and contexts.^12 13^ It is designed to assist decision-makers in setting investment priorities in a fair, transparent and systematic way.^11 12^ It uses the principle of collective opinion, or *wisdom of the crowds*, by querying experts and relevant stakeholders to generate and score research ideas against a predefined set of criteria.^14^ The output is a list and ranking of research priorities. The exercise is comprised of four key phases: (i) establishing an expert working group and defining scope, context and evaluation criteria, (ii) deciding on the weighting of the evaluation criteria, (iii) identifying context-specific experts for the generation and ranking of research questions against the proposed criteria and (iv) calculating research priority scores (RPS) and agreement between experts for each research idea.^11^ Below are the details for each phase of the Afghanistan research prioritisation exercise, which took place from April 2022 (experts identified) to February 2024 (survey data analysed), and is the first such exercise for Afghanistan based on a scoping of the published and grey literature.

### Phase 1: Establishing an expert working group and defining scope, context and evaluation criteria

In line with previous CHNRI exercises,^13 15 16^ a group of prominent experts on either health in Afghanistan or the CHNRI methodology was established to serve as the strategic advisory board (SAB). The purpose of the SAB was to provide guidance and input on key methodological decisions including: the scope of the exercise (short-term research priorities vs long-term research priorities), the context (focus on health research priorities vs health programming priorities, or both) and the criteria. Afghan and non-Afghan SAB members (n=8) with diverse health expertise in the Afghan context and/or CHRNI methodology were identified through related publications. Please see online supplemental table 1 for a list of SAB members, affiliations and expertise areas.

A survey was sent to the SAB to guide the establishment of the CHNRI criteria. An initial list of 13 criteria relevant to the Afghanistan context, gathered from other CHNRI exercises,^12^ were sent via email with instructions for ranking. Then the top five criteria were selected and specific criteria questions, or prompts, for each criterion were developed (table 1).

**Table 1:** Criteria and criteria questions for the Afghanistan research prioritisation exercise

Criterion	Criteria questions
Feasibility	Is it feasible to conduct this research considering cultural, financial, human resources, logistical and security challenges?
Effectiveness	Would the intervention that arises from the proposed research be effective in improving health outcomes in Afghanistan?
Equity	Can answering the question facilitate interventions that reduce population inequities (ie, to improve the health of the most vulnerable and disadvantaged)?
Answerability	Would you say it’s possible to design an ethically sound and implementable research study (eg, feasible, well-defined end points/outcomes, culturally appropriate) that can provide the requested answer?
Disease Burden Reduction	Would you say that interventions arising from the research would eventually contribute to a significant reduction in mortality / morbidity across the country?

### Phase 2: Weighting of the evaluation criteria

The study team decided to weigh all criteria equally in the analysis as it was determined that each criterion was of equal importance.

### Phase 3: Identifying experts for the generation and ranking of research questions against the proposed criteria

#### Expert identification

An initial researcher mapping exercise was conducted to identify researchers publishing three or more peer-reviewed articles on any health-related topic in Afghanistan from 2005 – 2022. These years were selected to maximise researcher response rate and ensure that researcher knowledge of Afghanistan was current. PubMed was selected as the primary database for this exercise given its vast library of public health-related scientific articles, its versatility in searching author publications and its credibility as a reputable biomedical and health search engine. A secondary database, Scopus, which covers a broader range of scientific fields, was used to verify the number of journal articles published by each identified researcher.

The following search strategy was used: ‘health AND Afghanistan’ and ‘NOT soldiers, Iraq, military, army’. Each resulting article was opened and explored to identify its authors. From there, each author’s hyperlinked page displaying their published articles was searched to determine whether they met the inclusion criteria of publishing at least three health-related, peer-reviewed articles on Afghanistan.

Once a researcher fulfilling the inclusion criteria was identified, their contact information, current or previous affiliations, number of health publications on Afghanistan and area(s) of expertise were recorded in an Excel spreadsheet. A total of 303 researchers were identified.

#### Research question generation

Identified researchers were provided instructions and requested to submit up to three priority health research questions for Afghanistan. Each submitted question was recorded in an Excel spreadsheet along with the researcher’s name and their self-identified area of expertise in health. In total, 96 researchers submitted 323 health research questions on Afghanistan.

#### Research question synthesis and survey creation

Research questions were organised by health topic area: health systems (n=135), maternal and child health and nutrition (n=84), communicable diseases (n=39), mental health and substance use (n=37) and miscellaneous (n=28). The health systems topic was selected for the first survey, given that it generated the highest number of questions. Related questions were reviewed, rephrased, split and combined, as required (while maintaining the essence of the questions) for the survey. The health systems research question synthesis occurred over several phases of editing and encompassed the use of an artificial intelligence tool, ChatGPT (V. 3.5), to help with the process of combining questions. For example, ChatGPT would be fed two submitted questions that were similar with a prompt telling it to combine the questions; the response would then be edited by the study team to come up with the final, synthesised question that was clear and reflected the essence of the original questions. A total of 53 questions were included in the final list of health systems research questions. A survey was then developed for researchers to score the final list of research questions against each priority-setting criterion (table 1).

All study data were collected and managed using Johns Hopkins University’s Research Electronic Data Capture (REDCap), a secure, web-based software platform designed to support data capture for research studies.^17 18^ REDCap allowed for setting automated, individualised reminders to the researchers, tracking survey completion and randomising question order per participant, among many other features.

#### Research question ranking / prioritisation

All identified researchers (n=303) were invited to take part in the ranking exercise, regardless of whether they had participated in question generation, and they self-selected to participate or not. A unique link to the survey was sent to each researcher through the REDCap server. Participants were asked to answer demographic questions and to score each of the 53 questions against the five criteria by answering: yes (one point), no (0 points) or undecided (0.5 points). If researchers felt they were not sufficiently informed to answer a particular question, they were given the option of answering with ‘insufficiently informed’. Questions were presented in a unique random order for each participant to minimise the impact of respondent-fatigue. Researchers were given 4 weeks to complete the ranking exercise and were sent weekly email reminders to complete the survey.

It should be noted that there are two other papers within this Supplement that report CHNRI results on the topics of (1) maternal and child health and nutrition and (2) communicable diseases in Afghanistan. These three CHNRI papers share an underlying origin that is the same up until the point of survey development, participant self-selection and research question ranking. Though the surveys on health systems, maternal and child health and nutrition, and communicable diseases were sent out to the same 303 researchers, they were each sent through separate surveys at different time points, with the health systems survey being sent close to a year prior to the next survey (maternal and child health and nutrition). Respondents self-selected to participate, meaning that the groups of researchers who responded to the three surveys may have some, but not complete, overlap.

### Phase 4: Calculating RPS

To determine the highest-ranking research priorities across survey respondents, the study team used two established CHNRI calculations: (i) the RPS and (ii) the average expert agreement (AEA). Two subgroup analyses were conducted— by Afghan versus non-Afghan and low- and middle-income country (LMIC) versus high-income country (HIC) respondents—to determine potential differences in priorities between respondent groups. Even if respondents of Afghan origin (ie, originally from Afghanistan) have lived or worked in a HIC for many years, there is still relevance to understanding whether their perspectives on research prioritisation differ from those who are not Afghan but undertake Afghan-related research, as they may have a closer connection to the country.

The RPS is the average score across participants and criteria; it provides the ‘collective optimism’ among scorers that a submitted research question fulfils the set criteria.^19^ It is a mean score calculated for each research question based on whether participants answered ‘Yes’ (one point), ‘No’ (0 points) or ‘Undecided’ (0.5 points). ‘Insufficiently informed’ responses were excluded from the calculation. Intermediate RPS ranging from 0%–100% were calculated for each criterion and an overall RPS for each research question was computed by taking a mean of the intermediate criteria scores. The higher the RPS, the higher its rank in terms of priority.^20^

The AEA illustrates the proportion of scorers selecting the most common score (mode) for each research question.^13^ It indicates the degree to which scorers are in consensus for each criterion.^13^ To calculate the AEA, the study team calculated the number of scorers who gave the most common score and divided it by the total number of respondents answering that question. An AEA of 75% and above indicates a high level of agreement between respondents.

Ranked research questions were categorised based on the CHNRI ‘4D’ framework,^19^ wherein questions are grouped into four key themes: Description, Delivery, Development and Discovery. For the purpose of this CHNRI, description questions focus on research assessing the burden of health problems in the population or describing/assessing the status of key health system factors; delivery questions focus on research that optimises the health status of the population through implementation research, operations research, health policy and systems research; development questions focus on research to improve already existing health interventions; and discovery questions focus on research that leads to innovations and new health interventions. Questions were further categorised into the overarching components of the Primary Healthcare Performance Initiative’s (PHCPI) conceptual framework (online supplemental figure 1): systems, inputs, service delivery, outputs and outcomes.^21^ This conceptual framework is specific to health systems,^22^ outlining the key inputs, core functions and desired goals of an effective primary healthcare system.^21^

The statistical software package STATA V.18 was used to conduct all data analyses.

## Results

### Characteristics of the respondents

We invited 303 researchers to score the research questions; 86 (28.3%) researchers partially completed the survey and 55 (18.2%) fully completed it; the non-response rate was 71.7%. Analyses of collective opinion exercises have found that responses for identifying the highest-ranking questions/ideas stabilise around 45–55 experts^20^; as such, we analysed answers from only those who fully completed the survey. Of the 55 respondents, over half were of Afghan origin (n=33, 60%), indicating that they were originally from Afghanistan, the majority were men (n=46, 84%), and about two-thirds were based in a HIC (n=35, 64%). Other researcher characteristics can be found in table 2.

**Table 2:** Characteristics among respondents who completed the survey

Characteristic	Proportion N (%)
Afghan	33 (60)
Non-Afghan	22 (30)
High-income country	35 (64)
Low or middle-income country	20 (36)
Men	46 (84)
Women	9 (16)
Years of experience in health research:	
1–3 years	0 (0)
4–7 years	9 (16)
8–10 years	8 (15)
11–15 years	16 (29)
16–20 years	9 (16)
20+	13 (24)
Years of health research experience related to Afghanistan:	
1–3 years	5 (9)
4–7 years	11 (20)
8–10 years	11 (20)
11–15 years	13 (24)
16–20 years	11 (20)
20+	4 (7)
Area of expertise (self-identified)*:	
Health systems	33 (60)
Maternal and child health	29 (53)
Communicable diseases	29 (53)
Nutrition	11 (20)
Sexual and reproductive health	11 (20)
Mental health	5 (9)
Non-communicablee diseases	10 (18)
Others	11 (20)
Place of employment*:	
Academic institution	23 (42)
Nongovernmental organisation	17 (31)
Within a healthcare setting	9 (16)
Donor agency	3 (5)
Other	10 (18)

* Respondents could have selected more than one option.

### Top research questions

The top 20 research priority questions according to the overall RPS are presented in table 3 (questions 1–10) and online supplemental table 2 (questions 11–20). The ranking of all questions scored is provided in online supplemental table 3. The RPS across the 53 questions ranged from 70% to 90%. The AEA scores across the top 20 questions were high (81% to 89%); they ranged from 69% to 89% across all questions, indicating a high level of agreement between the scorers. The highest priority questions were relatively diverse in terms of topic area, with questions spanning system-level factors, healthcare quality, community-based healthcare, improvements in the pharmaceutical sector, epidemiological trends, health management information systems and surveillance, access to care and approaches to improving service delivery in Afghanistan, among many others.

**Table 3:** Overall rank, intermediate research priority scores, overall research priority scores and average expert agreement for the top 10 research questions, including for Afghan and low- and middle-income country (LMIC) subgroups

Research question (Domain)	Expert group - ranking	Feasibility	Effectiveness	Equity	Answerability	Disease burden reduction	Overall Research Priority Score	Average Expert Agreement
What are health system factors preventing immunisation uptake at national and subnational levels in Afghanistan? (Delivery)	**Overall - 1st**	0.92	0.90	0.89	0.91	0.86	**89.95%**	**0.89**
	Afghan – 4^th^	0.92	0.92	0.91	0.95	0.86	**91.25%**	**0.91**
	LMIC – 7^th^	0.90	0.92	0.87	0.92	0.84	**89.05%**	**0.89**
What are the key challenges perceived by healthcare providers to providing high quality healthcare at primary, secondary and tertiary levels in Afghanistan? (Delivery)	**Overall - 2nd**	0.91	0.89	0.86	0.92	0.85	**88.54%**	**0.88**
	Afghan – 5^th^	0.89	0.89	0.86	0.95	0.89	**89.94%**	**0.90**
	LMIC – 1^st^	0.93	0.98	0.90	0.95	0.90	**92.95%**	**0.93**
What community-based healthcare package will serve the needs of Afghans today, especially in white areas* of the country (eg, CHWs, mobile health teams, family health houses)? (Delivery)	**Overall - 3rd**	0.90	0.86	0.88	0.88	0.90	**88.37%**	**0.88**
	Afghan – NA	–	–	–	–	–	–	–
	LMIC – NA	–	–	–	–	–	–	–
How can the pharmaceutical sector be strengthened to ensure access to affordable and quality medications in Afghanistan? (Development)	**Overall - 4th**	0.92	0.88	0.88	0.89	0.85	**88.30%**	**0.88**
	Afghan – 3^rd^	0.97	0.92	0.88	0.91	0.89	**91.37%**	**0.90**
	LMIC – 3^rd^	0.98	0.92	0.89	0.98	0.85	**92.20%**	**0.91**
What are the challenges and opportunities for improving the quality of BPHS/EPHS service delivery in Afghanistan? (Delivery)	**Overall - 5th**	0.92	0.87	0.84	0.89	0.83	**87.20%**	**0.86**
	Afghan – 6^th^	0.89	0.92	0.86	0.95	0.85	**89.64%**	**0.90**
	LMIC – 5^th^	0.95	0.93	0.88	0.95	0.80	**89.95%**	**0.89**
What are the levels of mortality, major morbidities and leading causes of death of children and adults in Afghanistan? (Description)	**Overall - 6th**	0.85	0.87	0.89	0.86	0.86	**86.44%**	**0.87**
	Afghan – 10^th^	0.86	0.89	0.89	0.89	0.89	**88.57%**	**0.89**
	LMIC – 2^nd^	0.88	0.95	0.95	0.90	0.95	**92.50%**	**0.93**
What are the challenges and solutions to consistent and accurate data collection and reporting within the health management information system? (Delivery)	**Overall - 7th**	0.89	0.85	0.84	0.89	0.82	**85.86%**	**0.85**
	Afghan – 7^th^	0.88	0.70	0.81	0.81	0.70	**89.48%**	**0.78**
	LMIC – 10^th^	0.90	0.90	0.85	0.90	0.87	**88.43%**	**0.88**
What is the level of access to basic health services in white areas* of Afghanistan? (Description)	**Overall - 8th**	0.85	0.86	0.88	0.89	0.80	**85.80%**	**0.86**
	Afghan – NA	–	–	–	–	–	–	–
	LMIC – 4^th^	0.89	0.94	0.91	0.89	0.92	**91.01%**	**0.91**
What approaches can improve tertiary healthcare service utilisation, quality, and patient satisfaction in Afghanistan? (Development)	**Overall - 9th**	0.91	0.88	0.78	0.89	0.82	**85.62%**	**0.84**
	Afghan – 9^th^	0.94	0.91	0.84	0.89	0.86	**88.81%**	**0.88**
	LMIC – 14^th^	0.89	0.88	0.88	0.90	0.85	**87.89%**	**0.88**
What are effective community-based strategies (eg, community-based nutrition package) for empowering and engaging communities in Afghanistan on healthy behaviours (eg, WASH, nutrition, vaccination, contraception) to reduce morbidity and improve survival? (Delivery)	**Overall - 10th**	0.86	0.85	0.83	0.86	0.88	**85.54%**	**0.85**
	Afghan – 2^nd^	0.87	0.66	0.91	0.79	0.79	**91.64%**	**0.81**
	LMIC – 9^th^	0.89	0.87	0.83	0.89	0.94	**88.60%**	**0.88**

* White areas: areas with no or limited access to basic health services.

BPHS/EPHS, Basic Package of Health Services (available at health facilities) and Essential Package of Hospital Services (available at hospitals); CHWs, community health workers; LMIC, low or middle-income country; NGO, non-governmental organisation; WASH, water, sanitation, and hygiene.

Half of the top 20 questions (n=10) were *delivery* questions, indicating a need for implementation and/or operational research into existing health system structures and service delivery approaches to optimise the health status of the Afghan population. These included the top three ranked questions focusing on health systems factors preventing immunisation uptake, key challenges in providing high quality healthcare as perceived by healthcare providers and community-based healthcare packages that would serve the needs of Afghans. Six of the top 20 questions (30%) were *development* questions, indicating a need for research into approaches for strengthening or modifying pre-existing and currently implemented interventions. These included the fourth-ranked question focused on strengthening the pharmaceutical sector and the ninth-ranked question focused on improving tertiary healthcare services utilisation, quality and patient satisfaction. Four of the top 20 questions (20%) were *description* questions focused on exploring the levels and leading causes of mortality and morbidity in children and adults in Afghanistan, the level of access to basic health services, the current availability and accessibility of core health system domains in Afghanistan’s primary healthcare system (eg, supply management, information systems and human resources), and the status of referral mechanisms between different levels of the healthcare system. No *discovery* questions were included in the list of 53 questions that were ranked.

When categorising the questions with PHCPI, a health systems-specific framework, nearly half of the top 20 research questions (n=9, 45%) were focused on the service delivery pillar. Service delivery questions included those that asked about challenges in providing high-quality healthcare, population health management, access to services and the availability of effective health interventions. About a third of the top questions (n=6, 30%) were systems-related questions, including those focused on health-financing, governance and leadership and adjustment to population health needs. The five highest priority questions were either service delivery or systems-related questions. Four questions were inputs-related, focusing on drugs and supplies, facility infrastructure, information systems, workforce and funds. Only one question was outcome-related—on the health status of children and adults in Afghanistan (question 6)—while none of the top 20 questions fell into the outputs pillar (please see online supplemental table 3 for PHCPI domains and subdomains of all scored questions).

The fifteen lowest scoring questions can be found in online supplemental table 4.

### Scores by nationality and income level

#### Afghan versus non-Afghan respondents

Of the Afghan respondents (n=33, 60%), the RPS and AEA for the top 20 questions ranged from 85% to 92% and 69% to 91%, respectively, indicating a high RPS and expert agreement. Many of the highest-ranking questions were the same as in the overall group but with a slightly different order (table 3). Similar to the results of the greater group, half of the top 20 questions were *delivery* questions (n=10), 7 were *development* questions (35%), 3 were *description* questions (15%) and none were *discovery* questions. Questions that are in table 4 (n=4) represent those that were not ranked in the top 20 among the greater group but were included in the Afghan respondents’ top 20. Overall, there was agreement between the Afghan and the non-Afghan respondents (n=22; 40%) in terms of the top 20 highest ranking questions (online supplemental table 5), though the order of ranking differed.

**Table 4:** Overall Rank, Intermediate Research Priority Scores, Overall Research Priority Scores and Average Expert Agreement for research questions that appeared in top 20 for Afghan or low- or middle-income country (LMIC) respondents, but not in the overall top 20

Ranking of research question within Sub-Group (Sub-Group)	Domain	Feasibility	Effectiveness	Equity	Answerability	Disease burden reduction	Overall Research Priority Score	Average Expert Agreement
What interventions can be implemented to strengthen the pharmaceutical sector in improving access to quality medications as well as to promote rational usage of medications in Afghanistan? (LMIC)	Development	0.88	0.89	0.82	0.92	0.92	**88.62%**	**0.89**
What community-based healthcare package will serve the needs of Afghanistan today, especially in white areas* of the country (eg, CHWs, mobile health teams, family health houses)? (Afghan)	Development	0.92	0.89	0.87	0.90	0.88	**89.14%**	**0.89**
How can the number and type of female healthcare professionals (eg, midwives, nurses, CHWs) be increased to address preferences in gender-based care among women? (LMIC)	Delivery	0.85	0.85	0.90	0.89	0.92	**88.20%**	**0.89**
What is the role of front-line healthcare professionals in ensuring high-quality healthcare delivery to Afghans? (LMIC)	Description	0.84	0.89	0.79	0.91	0.97	**88.15%**	**0.89**
How can the number and type of female healthcare professionals (eg, midwives, nurses, CHWs) be increased to address preferences in gender-based care among women? (Afghan)	Delivery	0.79	0.67	0.65	0.61	0.65	**87.30%**	**0.69**
What factors influence the motivation and productivity of community health workers in Afghanistan? (LMIC)	Delivery	0.90	0.93	0.85	0.88	0.84	**87.84%**	**0.87**
How should the BPHS/EPHS packages be revised in light of evolving disease burdens (eg, mental health, NCDs), population needs (eg, GBV) and health inequities in Afghanistan? (Afghan)	Development	0.87	0.74	0.90	0.92	0.90	**86.58%**	**0.86**
What type of community health worker programme (eg, training, tasks, pay structure) will optimise service coverage and reduce health inequities across Afghanistan? (LMIC)	Development	0.82	0.90	0.90	0.89	0.84	**86.94%**	**0.88**
What interventions can be implemented to strengthen the pharmaceutical sector in improving access to quality medications as well as to promote rational usage of medications in Afghanistan? (Afghan)	Development	0.89	0.89	0.79	0.88	0.84	**85.71%**	**0.85**

* White areas: areas with no or limited access to basic health services.

BPHS/EPHS, Basic Package of Health Services (available at health facilities) and Essential Package of Hospital Services (available at hospitals); CHWs, community health workers; GBV, gender-based violence; LMIC, low or middle-income country; NCDs, non-communicable diseases.

#### LMIC versus HIC respondents

Of the LMIC-based respondents (n=20, 36%), the RPS and AEA scores for the top 20 questions ranged from 86% to 93% and 87% to 93%, respectively, indicating a high RPS and expert agreement. Similar to the other subgroup analysis, many of the highest-ranking questions were the same as in the overall group, with a slightly different order (table 3). As with the greater group, nearly half of the top 20 questions were *delivery* questions (n=8), 7 were *development* questions (35%), 5 were *description* questions (20%), and none were *discovery* questions. Questions that are in table 4 (n=5) represent those that were not ranked in the top 20 among the greater group but were included in the LMIC respondents’ top 20. Generally, there was agreement between the LMIC and the non-LMIC respondents (n=35; 64%) in terms of the top 20 highest ranking questions (online supplemental table 6), though the order of ranking differed.

## Discussion

This exercise aimed to fill a critical gap in knowledge of health systems’ research priorities by generating consensus and establishing a research agenda for strengthening Afghanistan’s health system. To our knowledge, this exercise is the first systematic ranking of health research priorities for Afghanistan using the CHNRI methodology. Findings underscore the need to better understand how Afghanistan’s health system functions now and subsequent approaches for strengthening its key components. This is in line with what we expected, given resource constraints, health system fragility and the need to address immediate and practical healthcare challenges to achieve short- to medium-term benefits to population health. In line with this, ‘delivery’- and ‘development’- focused questions were prioritised compared with ‘discovery’ questions, demonstrating that participants assigned greater importance to more practical research questions that would explore features of and approaches to improving existing health system structures; a finding that has been mirrored in other CHNRI exercises in LMICs.^12^ The emerging themes focused on improving (1) service delivery (eg, availability of effective services, high quality healthcare, access, and population health management), (2) systems-level factors (eg, governance and leadership, health financing and population health needs), (3) inputs to the health system (eg, drugs and supplies, information systems, workforce and funds), and (4) outcomes (eg, health status) in Afghanistan. The top-ranking questions focused on: health systems factors preventing immunisation uptake; challenges in providing high quality healthcare; community-based health packages; approaches to strengthening the pharmaceutical sector and challenges and solutions for improving the quality of the Basic Package of Health Services (BPHS) and Essential Package of Hospital Services (EPHS) service delivery. We found that there was synergy among the subgroups examined (Afghan vs non-Afghan and LMIC vs non-LMIC respondents) in terms of the top 20 questions, underscoring consensus around the urgent need to address these research areas.

Overall, the highest-ranking question focused on health system-related barriers to immunisation uptake, which is likely due to Afghanistan’s historic struggle with achieving high immunisation coverage. While Afghanistan observed some gains in basic vaccination coverage rates throughout the Millennium Development Goals period (2000 – 2015),^9^ vaccination rates in Afghanistan remain low. In 2022–23, only 37% of children aged 12–23 months and 16% of children aged 24–35 months received all age-appropriate vaccinations, and there were wide inequalities between rural and urban children.^23 24^ Furthermore, Afghanistan is one of two countries in the world with endemic cases of polio. Compounded by the COVID-19 pandemic and suspension of polio vaccination campaigns in 2020, cases of wild poliovirus type 1 doubled from 29 in 2019 to 56 in 2020 and the country experienced an outbreak of circulating vaccine-derived poliovirus type 2 with 308 cases.^25^ This immunisation-related priority ranking is in line with CHNRI exercises conducted in neighbouring countries and similar low-income settings, including India^26^ and Ethiopia.^27^

Research on community-based packages of interventions and community engagement was also highly prioritised by researchers. Across Afghanistan, nearly 9.5 million people live in ‘white areas’ or areas in which people live at least 10 kilometers away from the nearest health facility.^28^ The training and deployment of nearly 30 000 volunteer community health workers through the BPHS has been necessary to provide health services to these rural communities and has contributed to health gains over the past two decades.^29^ However, challenges exist that threaten the motivation and the overall efficiency of this cadre,^29^ including funding and restrictions on female education, training, work and mobility, requiring implementation research to optimise the structure and efficiency of community-based packages given current realities and government restrictions. Descriptive studies to understand the current level of health service access are also necessary to identify vulnerable, hard-to-reach communities in order to plan for and allocate limited resources to community-based interventions aimed at reaching them. The high priority given to developing community-based strategies is in line with results from other CHNRI exercises across a range of health topics in humanitarian settings (eg, neonatal survival, reproductive, maternal, adolescent and child health, and treatment of child wasting), from neighbouring countries (India) and from other LMIC settings (Africa).^30^,^34^ Additionally, a recent CHNRI exercise for Somalia, another longstanding humanitarian setting, similarly revealed that understanding bottlenecks for delivering an essential package of health services in remote and hard-to-reach areas was of highest priority.^35^

### Implications and utility of research priorities

The research priorities that have emerged from this exercise are aligned with the strategic directions of the recently published Health Sector Transition Strategy 2023 – 2025,^36^ a product of the Health Sector Thematic Working Group (H-STWG) which was led by the WHO and UNICEF and included extensive consultations with the Ministry of Public Health, health professional bodies, health partners in Afghanistan and local non-governmental organisations. The strategy aims to ‘minimise avoidable morbidity and mortality in the short to medium term (2023–2025) by expanding the coverage and quality of health and nutrition services and strengthening health system resilience’.^37^ Serving as a document to guide coordinated health sector work for the international community in Afghanistan over this period, the document outlines four strategic directions: (i) strengthen and expand essential service coverage/utilisation and quality of care and improve financial risk protection for the most vulnerable groups, (ii) sustain and strengthen essential foundations of the health system that are necessary for meeting basic human health needs, (iii) strengthen the capacity to prevent, detect and respond to disease outbreaks and other health emergencies and (iv) strengthen harmonisation and alignment of financing toward national health priorities to increase predictability, adaptability and efficiency of funding.

It is worth noting that confirmation bias may have influenced the interpretation of results from this exercise as they align with the existing Health Sector Transition Strategy. Nonetheless, many identified research priorities do fit within the Strategy’s four strategic directions, especially the first. For example, under strategic direction 1, it is recommended to review the content of the BPHS/EPHS with a focus on improving efficiency and quality of care (research questions 5 and 13). Online supplemental table 7 outlines a mapping of research questions to strategic directions within the Health Sector Transition Strategy. If adequately funded and appropriately studied, findings from most of the top 20 identified research questions could be used to provide evidence-based guidance for both the medium-term activities under this strategy as well as for long-term strengthening of Afghanistan’s health system.

While addressing these research priorities will be essential to improve health system functioning from within Afghanistan, they could also be used to inform the funding and implementation of services by external partners, such as the WHO, UNICEF, United Nations Development Programme (UNDP), the World Bank and others. By identifying and filling evidence gaps, particularly those related to Delivery (for implementers) and Development (for funders), existing services, interventions and strategies could be strengthened to ultimately improve the provision of healthcare to a population in need. Of course, there are a multitude of barriers that must be overcome before priorities can be translated into action in the Afghan context, including those that are political, financial, social and human resource related. Focusing on research to answer critical questions is an appropriate first step.

### Strengths and limitations

This study has several strengths to note. First, it is timely, as there is an overdue need to facilitate efforts to bring Afghanistan closer to achieving its health-related SDG goals and international donors are now beginning to re-engage. Given the rapidly changing nature of international development/aid/funding, largely driven by the Trump administration in the United States, there is an even greater need for strategic and intentional resourcing. The methodology was robust, systematic and transparent, and we had diversity in our respondents (eg, origin, setting and profession) that further underscores the robust nature of our findings. Though the response rate was low (28%), 55 researchers fully completed the survey, meaning we reached the minimum number of experts needed to achieve stable scores (10).

In terms of limitations, there is the possibility for selection bias, given the self-selecting nature of participation and the fact that the researchers who fully completed the survey could be systematically different from those who did not. Certain groups may have been underrepresented; for example, only around 15% of respondents were women, which could lead to a bias of results whereby issues that are important or relevant to men are prioritised above those that are key for women. The same 303 researchers were invited to participate in all three surveys (health systems, maternal and child health and nutrition, and communicable diseases), though at different time points. While all were identified by their health-related publications through the initial research mapping exercise, it is plausible that one researcher may have responded to all three surveys while not necessarily being an ‘expert’ on all three topic areas. However, the response option of ‘insufficiently informed’ gave participants the option to not respond to a question if they did not feel informed enough to do so.

There was no involvement in the public throughout this work, and this study did not include representation from the Taliban. This runs the risk of neglecting the de facto government’s priorities and creating roadblocks when undertaking research within Afghanistan (where government permissions are required). As such, implementing the proposed research agenda could be challenging in practice, at least in the near term. Support from international partners could help to facilitate uptake of the proposed research given their longstanding role in the delivery of health services.

We received too many health systems research question submissions to include all within the survey (to avoid respondent fatigue), thus we may have lost some key questions that were not ranked. However, efforts were made to consolidate questions, where appropriate, and a subject matter expert was consulted prior to finalising the survey questionnaire. Similarly, there is a chance that the research ideas initially submitted did not capture all relevant and timely research questions relating to the health system in Afghanistan.

Finally, it should also be noted that the research questions were generated in August 2022 and scored in February 2024 given the many project activities that had to be completed before the scoring could take place (eg, the classification of research questions into sub-topics, review and consolidation of research questions, familiarisation with REDCap, creation of the survey and collection of responses). The evolving nature of the Taliban’s rulings, including the ban on midwifery and nursing training,^37^ and the shifting landscape related to international funding since then could have altered research priorities, making the current rankings less relevant. For example, today we might expect to see more research questions generated that relate to the gendered aspects of health service access or delivery.

## Conclusion

As Afghanistan’s first CHNRI exercise, this study provides a roadmap for both funding and implementing health systems research in Afghanistan on a short and long-term basis. However, we acknowledge that both health research and programming in Afghanistan will need to overcome challenges and will require contextual adaptation to be successful. Nevertheless, the results of this study provide donors with a transparent, systematically developed, evidence-based list of health systems research priorities for the country developed by Afghanistan health research experts. Filling these evidence gaps will result in further understanding of successful health strategies and interventions, ultimately suggesting possible means for strengthening the healthcare system in Afghanistan and improving the health status and lives of the Afghan people.

## Supplementary material

10.1136/bmjgh-2024-018578 online supplemental file 1

## Data Availability

All data relevant to the study are included in the article or uploaded as supplementary information.

## References

[1] UN Office for the Coordination of Humanitarian Affairs (2023). Afghanistan humanitarian response plan 2023. https://reliefweb.int/report/afghanistan/afghanistan-humanitarian-response-plan-2023-march-2023.

[2] Rights Watch H (2024). A disaster for the foreseeable future. http://www.hrw.org.

[3] United Nations Development Programme (UNDP) (2023). Afghanistan socio-economic outlook 2023. https://www.undp.org/afghanistan/publications/afghanistan-socio-economic-outlook-2023.

[4] Integrated Food Security Phase Classification (2024). Afghanistan: acute food insecurity situation for september - october 2024 and projection for november 2024 - march 2025. https://www.ipcinfo.org/ipc-country-analysis/details-map/en/c/1159434/?iso3=AFG.

[5] Food Security Information Network (FSIN) (2023). Global report on food crises 2023. www.fsinplatform.org/sites/default/files/resources/files/GRFC2023-hi-res.pdf.

[6] Office for the Coordination of Humanitarian Affairs (2023). Afghanistan: humanitarian update, february 2023. https://reliefweb.int/report/afghanistan/afghanistan-humanitarian-update-february-2023.

[7] UN Office for the Coordination of Humanitarian Affairs (2024). De facto authorities’ moral oversight in afghanistan: impacts on human rights. https://reliefweb.int/report/afghanistan/de-facto-authorities-moral-oversight-afghanistan-impacts-human-rights-july-2024-endarips.

[8] Alba S, Sondorp E, Kleipool E (2020). Estimating maternal mortality: what have we learned from 16 years of surveys in Afghanistan?. BMJ Glob Health.

[9] Akseer N, Salehi AS, Hossain SMM (2016). Achieving maternal and child health gains in Afghanistan: a Countdown to 2015 country case study. Lancet Glob Health.

[10] Safi N, Anwari P, Safi H (2022). Afghanistan’s health system under the Taliban: key challenges. The Lancet.

[11] Rudan I, Gibson JL, Ameratunga S (2008). Setting priorities in global child health research investments: guidelines for implementation of CHNRI method. Croat Med J.

[12] Rudan I, Yoshida S, Chan KY (2017). Setting health research priorities using the CHNRI method: VII. A review of the first 50 applications of the CHNRI method. J Glob Health.

[13] Frison S, Angood C, Khara T (2020). Prevention of child wasting: Results of a Child Health & Nutrition Research Initiative (CHNRI) prioritisation exercise. PLoS ONE.

[14] Wazny K, Chan KY, Collaborators CC (2018). Identifying potential uses of crowdsourcing in global health, conflict, and humanitarian settings: an adapted CHNRI (Child Health and Nutrition Initiative) exercise. J Glob Health.

[15] Arora NK, Mohapatra A, Gopalan HS (2017). Setting research priorities for maternal, newborn, child health and nutrition in India by engaging experts from 256 indigenous institutions contributing over 4000 research ideas: a CHNRI exercise by ICMR and INCLEN. J Glob Health.

[16] Irvine C, Armstrong A, Nagata JM (2018). Setting Global Research Priorities in Pediatric and Adolescent HIV Using the Child Health and Nutrition Research Initiative (CHNRI) Methodology. J Acquir Immune Defic Syndr.

[17] Harris PA, Taylor R, Thielke R (2009). Research electronic data capture (REDCap)--a metadata-driven methodology and workflow process for providing translational research informatics support. J Biomed Inform.

[18] Harris PA, Taylor R, Minor BL (2019). The REDCap consortium: Building an international community of software platform partners. J Biomed Inform.

[19] Rudan I (2016). Setting health research priorities using the CHNRI method: IV. Key conceptual advances. J Glob Health.

[20] Yoshida S, Rudan I, Cousens S (2016). Setting health research priorities using the CHNRI method: VI. Quantitative properties of human collective opinion. J Glob Health.

[21] Veillard J, Cowling K, Bitton A (2017). Better Measurement for Performance Improvement in Low- and Middle-Income Countries: The Primary Health Care Performance Initiative (PHCPI) Experience of Conceptual Framework Development and Indicator Selection. Milbank Q.

[22] Roberts M, Hsiao W, Berman P (2008). Getting Health Reform Right: A Guide to Improving Performance and Equity. Getting Health Reform Right: A Guide to Improving Performance and Equity.

[23] Watt A, Ministry of Public Health Wazir Akbar Khan A (2017). AFGHANISTAN demographic and health survey 2015 central statistics organization. http://cso.gov.af.

[24] United Nations Children’s Fund (UNICEF) (2023). Afghanistan Multiple Indicator Cluster Survey (MICS) 2022-2023, Summary Findings Report.

[25] Mohamed A, Akbar IE, Chaudhury S (2021). Progress Toward Poliomyelitis Eradication. MMWR Morb Mortal Wkly Rep.

[26] Wazny K, Arora NK, Mohapatra A (2019). Setting priorities in child health research in India for 2016-2025: a CHNRI exercise undertaken by the Indian Council for Medical Research and INCLEN Trust. J Glob Health.

[27] Korte ML, Teklie H, Taddesse L (2023). Setting a research agenda to advance maternal, newborn, and child health in Ethiopia: An adapted CHNRI prioritization exercise. J Glob Health.

[28] World Health Organization (2020). Underserved areas. https://dashboard.whe-him.org/index.php/maps-3/.

[29] Edward A, Branchini C, Aitken I (2015). Toward universal coverage in Afghanistan: A multi-stakeholder assessment of capacity investments in the community health worker system. Soc Sci Med.

[30] Mokashi T, Panigrahi S, Raman AV (2022). Priority Setting for Collaborative Health Systems Research in India: A Method and the Way Forward. J Health Manag.

[31] Kobeissi L, Nair M, Evers ES (2021). Setting research priorities for sexual, reproductive, maternal, newborn, child and adolescent health in humanitarian settings. Confl Health.

[32] Angood C, Kerac M, Black R (2021). Treatment of child wasting: results of a child health and nutrition research initiative (CHNRI) prioritisation exercise. F1000Res.

[33] Morof DF, Kerber K, Tomczyk B (2014). Neonatal survival in complex humanitarian emergencies: setting an evidence-based research agenda. Confl Health.

[34] Alobo M, Mgone C, Lawn J (2021). Research priorities in maternal and neonatal health in Africa: Results using the Child Health and Nutrition Research Initiative method involving over 900 experts across the continent. AAS Open Res.

[35] Ssendagire S, Mohamoud SA, Bashir F (2023). Health research prioritization in Somalia: setting the agenda for context specific knowledge to advance universal health coverage. Front Public Health.

[36] Global Financing Facility WHO (WHO), UNCF (UNICEF) (2023). Afghanistan health sector transition strategy 2023-2025. https://www.globalfinancingfacility.org/sites/default/files/Health%20Sector%20Transition%20Strategy%202023-2025.pdf.

[37] Nasari A, Marzouk S, Safi N (2025). A health emergency: Afghanistan’s nursing and midwifery ban. The Lancet.

