# Supplementary material for: Setting health systems research priorities for Afghanistan: an application of the child health and nutrition research initiative (CHNRI) methodology to set a roadmap to 2030
Source: BMJ Glob Health. 2025 Jul 25;10(Suppl 3):e018578. doi: 10.1136/bmjgh-2024-018578 (PMC12306304; doi:10.1136/bmjgh-2024-018578)

Supplemental Document

Table 1. Afghanistan CHNRI Strategic Advisory Board Members

SAB Member Name	Organization	Position Title	Health Topic Expertise	Number of Afghanistan Publications
Catherine Todd, MD, MPH	Pact	Senior Technical Advisor, Global Health	MNCH, HIV, SRH, mHealth	45
Hannah Tappis, MPH, DrPH	Jhpiego<br>Johns Hopkins University Bloomberg School of Public Health	Senior Measurement, Evaluation and Learning Advisor<br><br>Associate Faculty, Center for Humanitarian Health, Department of International Health	MNCH	23
David Peters, MD	York University, Faculty of Health	Dean	MNCH, Health Systems	21
Najibullah Safi, MD, MSc	World Health Organization	Program Manager, Health System Development	Health Systems, SRH, MNCH, Infectious Disease (COVID, Malaria)	18
Ahmad Shah Salehi, MD, MBA, MSc, PhD	Lapis Communications	Senior Public Health Advisor	MNCH, Health Systems	12
Kerri Wazny, PhD	Children's Investment Fund Foundation	EME Manager - Nutrition	CHNRI Methodology	-
Mickey Chopra, MD, MPH, PhD	World Bank	Global Solutions Lead for Service Delivery	MNCH, Health Systems, Mental Health	4
Robert Black, MD, MPH	Johns Hopkins University Bloomberg School of Public Health	Director for the Institute of International Programs<br><br>Professor	Health Systems, MNCH, Nutrition<br>CHRNI Methodology	-

Table 2. Overall Rank, Intermediate Research Priority Scores, Overall Research Priority Scores, and Average Expert Agreement for questions ranked 1-20, including for Afghan and low and middle-income country (LMIC) subgroups.

Ranking	Research Question	Domain (Description, Delivery, Development, Discovery)	PHCPI Domain	Average Expert Ranking	Feasibility	Effectiveness	Equity	Answerability	Disease Burden Reduction	Overall Research Priority Score	Average Expert Agreement
1	What are health system factors preventing immunization uptake at national and subnational levels in Afghanistan?	Delivery	System	Overall	0.92	0.90	0.89	0.91	0.86	**89.95%**	**0.89**
				**Afghan** - 4th	0.92	0.92	0.91	0.95	0.86	**91.25%**	**0.91**
				**LMIC** - 7th	0.90	0.92	0.87	0.92	0.84	**89.05%**	**0.89**
2	What are the key challenges perceived by healthcare providers to providing high quality healthcare at primary, secondary, and tertiary levels in Afghanistan?	Delivery	Service Delivery	Overall	0.91	0.89	0.86	0.92	0.85	**88.54%**	**0.88**
				**Afghan** - 5th	0.89	0.89	0.86	0.95	0.89	**89.94%**	**0.90**
				**LMIC** - 1st	0.93	0.98	0.90	0.95	0.90	**92.95%**	**0.93**
3	What community-based health care package will serve the needs of	Delivery	Service Delivery	Overall	0.90	0.86	0.88	0.88	0.90	**88.37%**	**0.88**

	Afghans today, especially in white areas[a] of the country (e.g., CHWs, mobile health teams, family health houses)?			**Afghan** N/A	-	-	-	-	-	**-**	**-**
				**LMIC** N/A	-	-	-	-	-	**-**	**-**
4	How can the pharmaceutical sector be strengthened to ensure access to affordable and quality medications in Afghanistan?	Development	System	Overall	0.92	0.88	0.88	0.89	0.85	**88.30%**	**0.88**
				**Afghan** – 3rd	0.97	0.92	0.88	0.91	0.89	**91.37%**	**0.90**
				**LMIC** - 3rd	0.98	0.92	0.89	0.98	0.85	**92.20%**	**0.91**
5	What are the challenges and opportunities for improving the quality of BPHS/EPHS service delivery in Afghanistan?	Delivery	Service Delivery	Overall	0.92	0.87	0.84	0.89	0.83	**87.20%**	**0.86**
				**Afghan** – 6th	0.89	0.92	0.86	0.95	0.85	**89.64%**	**0.90**
				**LMIC** - 5th	0.95	0.93	0.88	0.95	0.80	**89.95%**	**0.89**
6	What are the levels of mortality, major morbidities, and leading causes of death of children and adults in Afghanistan?	Description	Outcomes	Overall	0.85	0.87	0.89	0.86	0.86	**86.44%**	**0.87**
				**Afghan** – 10th	0.86	0.89	0.89	0.89	0.89	**88.57%**	**0.89**
				**LMIC** - 2nd	0.88	0.95	0.95	0.90	0.95	**92.50%**	**0.93**
7	What are the challenges and solutions to consistent and	Delivery	Inputs	Overall	0.89	0.85	0.84	0.89	0.82	**85.86%**	**0.85**

	accurate data collection and reporting within the health management information system?			**Afghan** – 7th	0.88	0.70	0.81	0.81	0.70	**89.48%**	**0.78**
				**LMIC** - 10th	0.90	0.90	0.85	0.90	0.87	**88.43%**	**0.88**
8	What is the level of access to basic health services in white areas[a] of Afghanistan?	Description	Service Delivery	Overall	0.85	0.86	0.88	0.89	0.80	**85.80%**	**0.86**
				**Afghan** N/A	-	-	-	-	-	**-**	**-**
				**LMIC** - 4th	0.89	0.94	0.91	0.89	0.92	**91.01%**	**0.91**
9	What approaches can improve tertiary healthcare service utilization, quality, and patient satisfaction in Afghanistan?	Development	Service Delivery	Overall	0.91	0.88	0.78	0.89	0.82	**85.62%**	**0.84**
				**Afghan** - 9th	0.94	0.91	0.84	0.89	0.86	**88.81%**	**0.88**
				**LMIC** - 14th	0.89	0.88	0.88	0.90	0.85	**87.89%**	**0.88**
10	What are effective community-based strategies (e.g., community-based nutrition package) for empowering and engaging	Delivery	Service Delivery	Overall	0.86	0.85	0.83	0.86	0.88	**85.54%**	**0.85**

	communities in Afghanistan on healthy behaviors (e.g., WASH, nutrition, vaccination, contraception) to reduce morbidity and improve survival?			**Afghan** - 2nd	0.87	0.66	0.91	0.79	0.79	**91.64%**	**0.81**
				**LMIC** - 9th	0.89	0.87	0.83	0.89	0.94	**88.60%**	**0.88**
11	What is the current status (availability and accessibility) of the core health system domains within Afghanistan's primary healthcare system (e.g., supply management, essential medicines, information systems, workforce)?	Description	Inputs	Overall	0.88	0.87	0.81	0.88	0.80	**84.90%**	**0.84**
				**Afghan** - 16th	0.89	0.90	0.81	0.90	0.82	**86.24%**	**0.86**
				**LMIC** - 6th	0.89	0.93	0.80	0.94	0.89	**89.06%**	**0.89**
12	What practical strategies can be implemented to strengthen human resources for health to improve service	Development	Inputs	Overall	0.88	0.82	0.82	0.87	0.82	**84.20%**	**0.83**
				**Afghan** - 20th	0.94	0.84	0.82	0.87	0.81	**85.47%**	**0.84**

	delivery and quality in Afghanistan?			**LMIC** - 17th	0.92	0.85	0.82	0.92	0.84	**87.00%**	**0.86**
13	What are the challenges and facilitators in implementing the BPHS/EPHS in regard to funding, management, implementation and evaluation in Afghanistan?	Delivery	System	Overall	0.89	0.84	0.81	0.87	0.80	**84.17%**	**0.83**
				**Afghan** - 1st	0.77	0.78	0.89	0.85	0.83	**92.25%**	**0.85**
				**LMIC** N/A	-	-	-	-	-	**-**	**-**
14	What are strategies for the effective and efficient management of humanitarian financial assistance to optimize health and health system outcomes, including in remote areas?	Delivery	System	Overall	0.83	0.85	0.83	0.85	0.84	**83.82%**	**0.84**
				**Afghan** - 13th	0.91	0.87	0.84	0.89	0.87	**87.38%**	**0.87**
				**LMIC** N/A	-	-	-	-	-	**-**	**-**
15	What is the status of referral mechanisms between different levels of the	Description	Service Delivery	Overall	0.85	0.84	0.81	0.88	0.81	**83.76%**	**0.84**
				**Afghan** - 18th	0.89	0.85	0.83	0.91	0.82	**85.92%**	**0.85**

	healthcare system (e.g., community, primary, secondary, tertiary healthcare)?			**LMIC** - 16th	0.87	0.93	0.87	0.89	0.82	**87.45%**	**0.88**
16	What are effective and sustainable strategies for improving communicable disease surveillance (e.g., polio, measles) to support timely investigation, diagnosis, response, and prevention in Afghanistan?	Development	Inputs	Overall	0.89	0.86	0.74	0.83	0.80	**82.43%**	**0.81**
				**Afghan** N/A	-	-	-	-	-	**-**	**-**
				**LMIC** - 20th	0.97	0.89	0.76	0.89	0.81	**86.49%**	**0.84**
17	What are the challenges and facilitators of the existing NGO contracting model in regard to funding, management, implementation and evaluation?	Delivery	System	Overall	0.89	0.83	0.78	0.88	0.74	**82.27%**	**0.81**
				**Afghan** – 17th	0.92	0.85	0.86	0.90	0.77	**86.02%**	**0.85**
				**LMIC** N/A	-	-	-	-	-	**-**	**-**

18	What approaches could improve patient satisfaction and trust within the healthcare system in Afghanistan?	Development	Service Delivery	Overall	0.87	0.81	0.83	0.86	0.75	**82.23%**	**0.81**
				**Afghan** – 11th	0.94	0.88	0.86	0.92	0.80	**87.83%**	**0.87**
				**LMIC** N/A	-	-	-	-	-	**-**	**-**
19	What strategies can Afghanistan and the international community employ to maintain and strengthen the health research capacity for Afghan researchers and health professionals?	Development	Inputs	Overall	0.87	0.82	0.77	0.88	0.76	**82.14%**	**0.81**
				**Afghan** N/A	-	-	-	-	-	**-**	**-**
				**LMIC** - 13th	0.92	0.85	0.82	0.95	0.86	**87.91%**	**0.87**
20	What are recommendations for donors and partners to improve health system governance and leadership mechanisms in Afghanistan at the national and subnational level?	Delivery	System	Overall	0.88	0.80	0.80	0.88	0.74	**81.94%**	**0.81**
				**Afghan** - 12th	0.89	0.88	0.91	0.92	0.80	**87.81%**	**0.88**
				**LMIC** - 19th	0.89	0.87	0.89	0.89	0.79	**86.61%**	**0.86**

Table 3. Overall Ranking, Research Question, 4D's Domain, PHCPI Domain and Subdomain, Intermediate Research Priority Scores, Overall Research Priority Scores, and Average Expert Agreement for all scored research questions

**Ranking**	**Research Question**	**Domain (Description, Delivery, Development, Discovery)**	**PHCPI Domain**	**PHCPI Sub-Domain**	**Feasibility**	**Effectiveness**	**Equity**	**Answerability**	**Disease Burden Reduction**	**Overall RPS**	**AEA**
1	What are health system factors preventing immunization uptake at national and subnational levels in Afghanistan?	Delivery	System		0.9231	0.9038	0.8942	0.9135	0.8627	**89.95%**	**0.89**
2	What are the key challenges perceived by healthcare providers to providing high quality healthcare at primary, secondary, and tertiary levels in Afghanistan?	Delivery	Service Delivery	Availability of Effective PHC Services; High Quality Primary Health Care	0.9074	0.8889	0.8611	0.9151	0.8545	**88.54%**	**0.88**
3	What community-based health care package will serve the needs of Afghans today, especially in white areas of the country (e.g., CHWs, mobile health teams, family health houses)?	Delivery	Service Delivery	Population Health Management	0.8980	0.8627	0.8800	0.8776	0.9000	**88.37%**	**0.88**
4	How can the pharmaceutical sector be strengthened to ensure access to affordable and quality medications in Afghanistan?	Development	System	Adjustment to Population Health Needs	0.9200	0.8800	0.8750	0.8900	0.8500	**88.30%**	**0.88**

5	What are the challenges and opportunities for improving the quality of BPHS/EPHS service delivery in Afghanistan?	Delivery	Service Delivery	High Quality Primary Health Care	0.9245	0.8704	0.8396	0.8922	0.8333	**87.20%**	**0.86**
6	What are the levels of mortality, major morbidities, and leading causes of death of children and adults in Afghanistan?	Description	Outcomes	Health Status	0.8529	0.8679	0.8868	0.8558	0.8585	**86.44%**	**0.87**
7	What are the challenges and solutions to consistent and accurate data collection and reporting within the health management information system (HMIS)?	Delivery	Inputs	Information Systems	0.8889	0.8519	0.8400	0.8889	0.8235	**85.86%**	**0.85**
8	What is the level of access to basic health services in white areas of Afghanistan?	Description	Service Delivery	Access	0.8542	0.8600	0.8800	0.8922	0.8039	**85.80%**	**0.86**
9	What approaches can improve tertiary healthcare service utilization, quality, and patient satisfaction in Afghanistan?	Development	Service Delivery	Availability of Effective PHC Services; High Quality Primary Health Care	0.9135	0.8774	0.7788	0.8942	0.8173	**85.62%**	**0.84**

10	What are effective community-based strategies (e.g., community-based nutrition package) for empowering and engaging communities in Afghanistan on healthy behaviors (e.g., WASH, nutrition, vaccination, contraception) to reduce morbidity and improve survival?	Delivery	Service Delivery	Population Health Management	0.8627	0.8491	0.8269	0.8558	0.8824	**85.54%**	**0.85**
11	What is the current status (availability and accessibility) of the core health system domains within Afghanistan's primary healthcare system (e.g., supply management/essential medicines, information systems, workforce)?	Description	Inputs	Drugs & Supplies ; Facility Infrastructure; information systems; workforce ; funds	0.8750	0.8725	0.8113	0.8824	0.8039	**84.90%**	**0.84**
12	What practical strategies can be implemented to strengthen human resources for health to improve service delivery and quality in Afghanistan?	Development	Inputs	Workforce	0.8824	0.8241	0.8173	0.8654	0.8208	**84.20%**	**0.83**
13	What are the challenges and facilitators in implementing the BPHS/EPHS in regard to funding, management, implementation and evaluation in Afghanistan?	Delivery	System		0.8868	0.8396	0.8137	0.8725	0.7959	**84.17%**	**0.83**

14	What are strategies for the effective and efficient management of humanitarian financial assistance to optimize health and health system outcomes, including in remote areas?	Delivery	System	Health Financing	0.8261	0.8469	0.8333	0.8478	0.8367	**83.82%**	**0.84**
15	What is the status of referral mechanisms between different levels of the healthcare system (e.g., community, primary, secondary, tertiary healthcare)?	Description	Service Delivery	High Quality Primary Health Care	0.8491	0.8426	0.8113	0.8774	0.8077	**83.76%**	**0.84**
16	What are effective and sustainable strategies for improving communicable disease surveillance (e.g., polio, measles) to support timely investigation, diagnosis, response, and prevention in Afghanistan?	Development	Inputs	Information Systems	0.8922	0.8558	0.7404	0.8333	0.8000	**82.43%**	**0.81**
17	What are the challenges and facilitators of the existing NGO contracting model in regard to funding, management, implementation and evaluation?	Delivery	System	Health Financing	0.8900	0.8269	0.7788	0.8774	0.7404	**82.27%**	**0.81**
18	What approaches could improve patient satisfaction and trust within the healthcare system in Afghanistan?	Development	Service Delivery	Availability of Effective PHC Services	0.8700	0.8077	0.8302	0.8585	0.7451	**82.23%**	**0.81**

19	What strategies can Afghanistan and the international community employ to maintain and strengthen the health research capacity for Afghan researchers and health professionals?	Development	Inputs	Workforce	0.8725	0.8208	0.7692	0.8846	0.7600	**82.14%**	**0.81**
20	What are recommendations for donors and partners to improve health system governance and leadership mechanisms in Afghanistan at the national and subnational level?	Delivery	System	Governance & Leadership	0.8750	0.8019	0.8019	0.8774	0.7407	**81.94%**	**0.81**
21	How can the number and type of female healthcare professionals (e.g., midwives, nurses, CHWs) be increased to address preferences in gender-based care among women?	Delivery	Inputs	Workforce	0.7600	0.8208	0.8333	0.7766	0.8462	**80.74%**	**0.82**
22	How should the BPHS/EPHS packages be revised in light of evolving disease burdens (e.g., mental health, NCDs), population needs (e.g., GBV) and health inequities in Afghanistan?	Development	System	Adjustment to Population Health Needs	0.8173	0.7308	0.8365	0.8208	0.8300	**80.71%**	**0.81**

23	What is the role of front-line healthcare professionals in ensuring high-quality healthcare delivery to Afghans?	Description	Inputs	Workforce	0.8396	0.8077	0.7451	0.8529	0.7857	**80.62%**	**0.80**
24	What factors influence the motivation and productivity of community health workers (CHWs) in Afghanistan?	Delivery	Inputs	Workforce	0.8585	0.8000	0.7685	0.8333	0.7685	**80.58%**	**0.80**
25	What interventions can be implemented to strengthen the pharmaceutical sector in improving access to quality medications as well as to promote rational usage of medications in Afghanistan?	Development	Inputs	Drugs & Supplies	0.8333	0.8173	0.7400	0.8173	0.8077	**80.31%**	**0.80**
26	What type of community health worker program (e.g., training, tasks, pay structure) will optimize service coverage and reduce health inequities across Afghanistan?	Development	Service Delivery	Population Health Management	0.7755	0.8269	0.8462	0.8039	0.7547	**80.14%**	**0.81**
27	What are the enablers, barriers, and strategies for female health workers to provide high quality services in urban compared to rural settings of Afghanistan?	Delivery	Service Delivery	High Quality Primary Health Care	0.7075	0.8077	0.8019	0.8039	0.8208	**78.84%**	**0.80**

28	How can health information systems across the country be strengthened to improve data quality and data use for tracking population health outcomes?	Development	Inputs	Information Systems	0.8491	0.7500	0.7778	0.8426	0.7170	**78.73%**	**0.77**
29	What are the challenges and solutions for effective PHC delivery (e.g., governance/leadership, health financing) in Afghanistan presently compared to the past 20 years?	Delivery	System	Governance & Leadership; Health Financing; Adjustment to Population Health Needs	0.8077	0.8056	0.7736	0.7870	0.7364	**78.20%**	**0.78**
30	How can the imbalances in human resources for health (e.g., skills/capacity, gender, and urban/rural disparities) be addressed to ensure optimal healthcare delivery in Afghanistan?	Delivery	Inputs	Workforce	0.7692	0.7981	0.7843	0.7404	0.7885	**77.61%**	**66.80**
31	What are the challenges and solutions to address the health commodity shortages in the health system of Afghanistan (e.g., social franchising, networked providers, long term framework)?	Delivery	System		0.7955	0.7784	0.7635	0.8073	0.7492	**77.88%**	**0.78**
32	What proportion of the current healthcare provider workforce is female and how has this changed in recent years?	Description	Inputs	Workforce	0.7913	0.7746	0.7599	0.8039	0.7453	**77.50%**	**0.77**

33	What are the most effective and sustainable models of contracting NGOs to deliver healthcare services in Afghanistan (e.g., lumpsum, pay-for-performance, incentives)?	Delivery	System	Health Financing	0.7871	0.7708	0.7564	0.8005	0.7415	**77.13%**	**0.77**
34	What strategies are currently in place to ensure the continued compensation of healthcare workers in Afghanistan, and how effective are they?	Description / Delivery	System	Health Financing	0.7828	0.7670	0.7528	0.7971	0.7376	**76.75%**	**0.76**
35	What strategies can be effective to leverage the existing humanitarian platform to expand vertical program interventions delivery?	Development	Service Delivery	Facility Organization & Management	0.7786	0.7633	0.7492	0.7937	0.7338	**76.37%**	**0.76**
36	What is the updated demographic composition (e.g., age, sex, employment, income, marital status, ethnicity) of the Afghan population at the national and subnational levels?	Description	Outcomes	Health Status	0.7744	0.7595	0.7457	0.7903	0.7299	**76.00%**	**0.76**
37	What are feasible and effective methods to strengthen the overall healthcare infrastructure (e.g., facilities, equipment, medications, WASH in facilities, e-health systems) in urban and rural areas in Afghanistan?	Development	Inputs	Drugs & Supplies ; Facility Infrastructure; information systems; workforce ; funds	0.7701	0.7557	0.7421	0.7870	0.7261	**75.62%**	**0.75**

38	What are feasible and reliable innovations for implementing civil registration and vital statistics (CRVS) systems in Afghanistan (e.g., sample registration systems)?	Development	Inputs	Information Systems	0.7659	0.7519	0.7385	0.7836	0.7222	**75.24%**	**0.75**
39	What are strategies to ensure appropriate usage of medicines (e.g., dose, amount) in the current resource restricted environment in Afghanistan?	Delivery	Inputs	Drugs & Supplies	0.7617	0.7481	0.7349	0.7802	0.7184	**74.87%**	**0.75**
40	What are the most sustainable and effective healthcare financing models (e.g., domestic solutions, private sector involvement, ARTF/trust funds) to support primary, secondary/tertiary care in Afghanistan?	Delivery	System	Health Financing	0.7574	0.7444	0.7314	0.7768	0.7145	**74.49%**	**0.74**
41	What is the impact of the recent political and economic changes on out-of-pocket expenditure on health among Afghans?	Description	System	Health Financing	0.7532	0.7406	0.7278	0.7734	0.7106	**74.11%**	**0.74**
42	What is Afghanistan's level of preparedness in responding to external shocks (e.g., political changes, pandemics, natural disasters) and what are approaches for improving the healthcare system's	Description / Development	Outcomes	Resilience of Health Systems	0.7490	0.7368	0.7242	0.7700	0.7068	**73.74%**	**0.74**

	resilience to manage such shocks?											
43	What were the challenges and successes of Afghanistan’s health system governance from 2001 to 2021?	Description	System	Governance & Leadership	0.7447	0.7330	0.7207	0.7666	0.7029	**73.36%**	**0.73**
44	How does Afghanistan's healthcare system (public and private) respond to the needs of patients who may require treatment options outside the country?	Description	Outcomes	Responsiveness to People	0.7405	0.7292	0.7171	0.7632	0.6991	**72.98%**	**0.73**
45	What is the impact of Afghanistan's current health service delivery model (post-August 2021) on health outcomes?	Description	Outcomes	Health Status	0.7363	0.7255	0.7135	0.7598	0.6952	**72.60%**	**0.72**
46	What is the current status of Afghanistan’s health system governance (e.g., availability, relevance, and capacity)?	Description	System	Governance & Leadership	0.7320	0.7217	0.7100	0.7564	0.6914	**72.23%**	**0.72**

47	What are effective and sustainable opportunities for digital health innovation (e.g., mobile mentoring, monitoring, and telemedicine) to improve health outcomes in rural and urban areas?	Development	Service Delivery	High Quality Primary Health Care	0.7278	0.7179	0.7064	0.7530	0.6875	**71.85%**	**0.72**
48	What are effective approaches (e.g., cost-recovery mechanisms such as user fees or premiums) to mobilizing domestic funds to self-sustain the healthcare system in Afghanistan, and what are the strengths and weaknesses of each approach?	Development	System	Health Financing	0.7236	0.7141	0.7028	0.7496	0.6836	**71.47%**	**0.71**
49	What are the opportunities to alleviate economic sanctions and continue official development assistance to Afghanistan?	Delivery	System	Health Financing	0.7193	0.7103	0.6993	0.7462	0.6798	**71.10%**	**0.71**
50	What measures can Afghanistan implement to ensure the sustainability of its technical expertise in health economics and healthcare financing?	Delivery	Inputs	Workforce	0.7151	0.7066	0.6957	0.7428	0.6759	**70.72%**	**0.71**
51	What are strategies for mitigating the impact of the brain drain of healthcare professionals on health service coverage and	Delivery	Inputs	Workforce	0.7108	0.7028	0.6921	0.7394	0.6721	**70.34%**	**0.70**

	overall functionality of the health system?									
52	What are innovative and context adapted approaches (e.g., insurance schemes) for reducing the high out-of-pocket expenditure on health in Afghanistan?	Delivery	System	Health Financing	0.7066	0.6990	0.6886	0.7360	0.6682	**69.97%**	**0.70**
53	What are effective strategies for ensuring safety and security (e.g., physical, emotional) for patients and healthcare providers in Afghanistan?	Delivery	Service Delivery	Availability of Effective PHC Services	0.7024	0.6952	0.6850	0.7326	0.6644	**69.59%**	**0.69**

Table 4. Overall Rank, Intermediate Research Priority Scores, Overall Research Priority Scores, and Average Expert Agreement for the lowest 15 research questions

**Ranking**	**Research Question**	**Domain (Description, Delivery, Development, Discovery)**	**PHCPI Domain**	**Feasibility**	**Effectiveness**	**Equity**	**Answerability**	**Disease Burden Reduction**	**Overall RPS**	**AEA**
38	What are feasible and reliable innovations for implementing	Development	Inputs	0.7659	0.7519	0.7385	0.7836	0.7222	**75.24%**	**0.75**

	civil registration and vital statistics (CRVS) systems in Afghanistan (e.g., sample registration systems)?									
39	What are strategies to ensure appropriate usage of medicines (e.g., dose, amount) in the current resource restricted environment in Afghanistan?	Delivery	Inputs	0.7617	0.7481	0.7349	0.7802	0.7184	**74.87%**	**0.75**
40	What are the most sustainable and effective healthcare financing models (e.g., domestic solutions,	Delivery	System	0.7574	0.7444	0.7314	0.7768	0.7145	**74.49%**	**0.74**

	private sector involvement, ARTF/trust funds) to support primary, secondary/tertiary care in Afghanistan?									
41	What is the impact of the recent political and economic changes on out-of-pocket expenditure on health among Afghans?	Description	System	0.7532	0.7406	0.7278	0.7734	0.7106	**74.11%**	**0.74**
42	What is Afghanistan's level of preparedness in responding to external shocks (e.g., political changes, pandemics, natural disasters) and what are	Description / Development	Outcomes	0.7490	0.7368	0.7242	0.7700	0.7068	**73.74%**	**0.74**

	approaches for improving the healthcare system’s resilience to manage such shocks?									
43	What were the challenges and successes of Afghanistan’s health system governance from 2001 to 2021?	Description	System	0.7447	0.7330	0.7207	0.7666	0.7029	**73.36%**	**0.73**
44	How does Afghanistan's healthcare system (public and private) respond to the needs of patients who may require treatment options outside the country?	Description	Outcomes	0.7405	0.7292	0.7171	0.7632	0.6991	**72.98%**	**0.73**

45	What is the impact of Afghanistan's current health service delivery model (post-August 2021) on health outcomes?	Description	Outcomes	0.7363	0.7255	0.7135	0.7598	0.6952	**72.60%**	**0.72**
46	What is the current status of Afghanistan's health system governance (e.g., availability, relevance, and capacity)?	Description	System	0.7320	0.7217	0.7100	0.7564	0.6914	**72.23%**	**0.72**
47	What are effective and sustainable opportunities for digital health innovation (e.g., mobile mentoring, monitoring, and telemedicine) to improve	Development	Service Delivery	0.7278	0.7179	0.7064	0.7530	0.6875	**71.85%**	**0.72**

	health outcomes in rural and urban areas?									
48	What are effective approaches (e.g., cost-recovery mechanisms such as user fees or premiums) to mobilizing domestic funds to self-sustain the healthcare system in Afghanistan, and what are the strengths and weaknesses of each approach?	Development	System	0.7236	0.7141	0.7028	0.7496	0.6836	**71.47%**	**0.71**
49	What are the opportunities to alleviate economic sanctions and continue	Delivery	System	0.7193	0.7103	0.6993	0.7462	0.6798	**71.10%**	**0.71**

	official development assistance to Afghanistan?									
50	What measures can Afghanistan implement to ensure the sustainability of its technical expertise in health economics and healthcare financing?	Delivery	Inputs	0.7151	0.7066	0.6957	0.7428	0.6759	**70.72%**	**0.71**
51	What are strategies for mitigating the impact of the brain drain of healthcare professionals on health service coverage and overall functionality of the health system?	Delivery	Inputs	0.7108	0.7028	0.6921	0.7394	0.6721	**70.34%**	**0.70**

52	What are innovative and context adapted approaches (e.g., insurance schemes) for reducing the high out-of-pocket expenditure on health in Afghanistan?	Delivery	System	0.7066	0.6990	0.6886	0.7360	0.6682	**69.97%**	**0.70**
53	What are effective strategies for ensuring safety and security (e.g., physical, emotional) for patients and healthcare providers in Afghanistan?	Delivery	Service Delivery	0.7024	0.6952	0.6850	0.7326	0.6644	**69.59%**	**0.69**

Table 5. Overall Rank, Intermediate Research Priority Scores, Overall Research Priority Scores, and Average Expert Agreement for top 20 research questions of non-Afghan respondents

Ranking	Research Question	Domain (Description, Delivery, Development, Discovery)	Feasibility	Effectiveness	Equity	Answerability	Disease Burden Reduction	Overall RPS	AEA
1	What are health system factors preventing immunization uptake at national and subnational levels in Afghanistan?	Delivery	0.93	0.88	0.88	0.85	0.87	**87.87%**	**0.87**
2	What is the level of access to basic health services in white areas of Afghanistan?	Description	0.91	0.82	0.86	0.97	0.81	**87.37%**	**0.87**
3	What community-based health care package will serve the needs of Afghans today, especially in white areas of the country (e.g., CHWs, mobile health teams, family health houses)?	Development	0.87	0.82	0.90	0.83	0.93	**87.04%**	**0.87**
4	What are the key challenges perceived by healthcare providers to providing high quality healthcare at primary, secondary, and tertiary levels in Afghanistan?	Delivery	0.93	0.88	0.86	0.86	0.80	**86.52%**	**0.85**

5	What are the challenges and opportunities for improving the quality of BPHS/EPHS service delivery in Afghanistan?	Delivery	0.98	0.79	0.80	0.80	0.81	**83.40%**	**0.81**
6	What is the current status (availability and accessibility) of the core health system domains within Afghanistan's primary healthcare system (e.g., supply management/essential medicines, information systems, workforce)?	Description	0.86	0.84	0.82	0.85	0.79	**83.04%**	**0.83**
7	What are the levels of mortality, major morbidities, and leading causes of death of children and adults in Afghanistan?	Description	0.84	0.82	0.88	0.80	0.81	**83.03%**	**0.83**
8	How can the pharmaceutical sector be strengthened to ensure access to affordable and quality medications in Afghanistan?	Development	0.83	0.82	0.86	0.86	0.78	**82.98%**	**0.83**

9	What are effective and sustainable strategies for improving communicable disease surveillance (e.g., polio, measles) to support timely investigation, diagnosis, response, and prevention in Afghanistan?	Development	0.85	0.88	0.75	0.82	0.82	**82.50%**	**0.82**
10	What practical strategies can be implemented to strengthen human resources for health to improve service delivery and quality in Afghanistan?	Development	0.80	0.80	0.82	0.86	0.84	**82.23%**	**0.83**
11	What approaches can improve tertiary healthcare service utilization, quality, and patient satisfaction in Afghanistan?	Development	0.88	0.82	0.68	0.90	0.75	**80.50%**	**0.79**
12	What are the challenges and solutions to consistent and accurate data collection and reporting within the health management information system (HMIS)?	Delivery	0.86	0.76	0.76	0.86	0.79	**80.48%**	**0.79**

13	What is the status of referral mechanisms between different levels of the healthcare system (e.g., community, primary, secondary, tertiary healthcare)?	Description	0.79	0.83	0.79	0.83	0.79	**80.48%**	**0.81**
14	What strategies can Afghanistan and the international community employ to maintain and strengthen the health research capacity for Afghan researchers and health professionals?	Development	0.90	0.80	0.75	0.85	0.71	**80.21%**	**0.78**
15	What are the challenges and solutions for effective PHC delivery (e.g., governance/leadership, health financing) in Afghanistan presently compared to the past 20 years?	Delivery	0.81	0.79	0.84	0.79	0.73	**78.98%**	**0.79**
16	What are strategies for the effective and efficient management of humanitarian financial assistance to optimize health and health system	Delivery	0.71	0.82	0.82	0.79	0.79	**78.61%**	**0.80**

	outcomes, including in remote areas?								
17	What are the challenges and facilitators of the existing NGO contracting model in regard to funding, management, implementation and evaluation?	Delivery	0.85	0.79	0.65	0.84	0.70	**76.62%**	**0.75**
18	**What is the updated demographic composition (e.g., age, sex, employment, income, marital status, ethnicity) of the Afghan population at the national and subnational levels?**	Description	0.83	0.68	0.80	0.85	0.65	**76.41%**	**0.75**
19	What are effective community-based strategies (e.g., community-based nutrition package) for empowering and engaging communities in Afghanistan on healthy behaviors (e.g., WASH, nutrition, vaccination, contraception) to	Delivery	0.73	0.79	0.79	0.71	0.81	**76.40%**	**0.77**

	reduce morbidity and improve survival?								
20	**What are the challenges and solutions to address the health commodity shortages in the health system of Afghanistan (e.g., social franchising, networked providers, long term framework)?**	Delivery	0.76	0.76	0.76	0.75	0.75	**75.82%**	**0.76**

***Bold indicates that questions were not included in the top twenty ranked questions among the overall group.**

Table 6. Overall Rank, Intermediate Research Priority Scores, Overall Research Priority Scores, and Average Expert Agreement for top 20 research questions of respondents in high income countries

**Ranking**	**Research Question**	**Domain (Description, Delivery, Development, Discovery)**	**Feasibility**	**Effectiveness**	**Equity**	**Answerability**	**Disease Burden Reduction**	**Overall RPS**	**AEA**
1	What community-based health care package will serve the needs of Afghans today, especially in white areas of the country (e.g., CHWs, mobile health	Development	0.94	0.88	0.94	0.91	0.92	**91.64%**	**0.91**

	teams, family health houses)?								
2	What are health system factors preventing immunization uptake at national and subnational levels in Afghanistan?	Delivery	0.94	0.89	0.91	0.91	0.88	**90.49%**	**0.90**
3	What are the key challenges perceived by healthcare providers to providing high quality healthcare at primary, secondary, and tertiary levels in Afghanistan?	Delivery	0.90	0.84	0.84	0.90	0.83	**85.98%**	**0.85**
4	How can the pharmaceutical sector be strengthened to ensure access to affordable and quality medications in Afghanistan?	Development	0.88	0.85	0.87	0.83	0.85	**85.76%**	**0.85**
5	What are the challenges and opportunities for improving the quality of BPHS/EPHS service delivery in Afghanistan?	Delivery	0.91	0.84	0.82	0.86	0.85	**85.56%**	**0.84**
6	What are strategies for the effective and efficient management of humanitarian financial assistance to optimize health and health system	Delivery	0.83	0.85	0.85	0.86	0.85	**84.91%**	**0.85**

	outcomes, including in remote areas?								
7	What are the challenges and solutions to consistent and accurate data collection and reporting within the health management information system (HMIS)?	Delivery	0.88	0.82	0.83	0.88	0.80	**84.37%**	**0.84**
8	What approaches can improve tertiary healthcare service utilization, quality, and patient satisfaction in Afghanistan?	Development	0.92	0.88	0.72	0.89	0.80	**84.19%**	**0.83**
9	What are effective community-based strategies (e.g., community-based nutrition package) for empowering and engaging communities in Afghanistan on healthy behaviors (e.g., WASH, nutrition, vaccination, contraception) to reduce morbidity and improve survival?	Delivery	0.84	0.84	0.82	0.84	0.85	**83.84%**	**0.84**

10	What are the challenges and facilitators of the existing NGO contracting model in regard to funding, management, implementation and evaluation?	Delivery	0.89	0.83	0.79	0.90	0.78	**83.77%**	**0.83**
11	What are the challenges and facilitators in implementing the BPHS/EPHS in regard to funding, management, implementation and evaluation in Afghanistan?	Delivery	0.90	0.79	0.80	0.89	0.77	**83.08%**	**0.82**
12	What is the level of access to basic health services in white areas of Afghanistan?	Description	0.83	0.81	0.86	0.89	0.74	**82.92%**	**0.83**
13	What are the levels of mortality, major morbidities, and leading causes of death of children and adults in Afghanistan?	Description	0.84	0.82	0.85	0.83	0.80	**82.73%**	**0.83**
14	What practical strategies can be implemented to strengthen human resources for health to improve service delivery and quality in Afghanistan?	Development	0.86	0.81	0.82	0.83	0.81	**82.57%**	**0.82**

15	What is the current status (availability and accessibility) of the core health system domains within Afghanistan’s primary healthcare system (e.g., supply management/essential medicines, information systems, workforce)?	Description	0.86	0.84	0.82	0.85	0.76	**82.50%**	**0.82**
16	What is the status of referral mechanisms between different levels of the healthcare system (e.g., community, primary, secondary, tertiary healthcare)?	Description	0.84	0.79	0.78	0.87	0.80	**81.65%**	**0.81**
17	What approaches could improve patient satisfaction and trust within the healthcare system in Afghanistan?	Development	0.85	0.78	0.82	0.82	0.77	**80.87%**	**0.80**
18	What are effective and sustainable strategies for improving communicable disease surveillance (e.g., polio, measles) to support timely investigation, diagnosis, response, and prevention in Afghanistan?	Development	0.85	0.83	0.73	0.80	0.80	**80.18%**	**0.79**

19	**What are the challenges and solutions for effective PHC delivery (e.g., governance/leadership, health financing) in Afghanistan presently compared to the past 20 years?**	Delivery	0.82	0.81	0.81	0.81	0.74	**79.97%**	**0.80**
20	What are recommendations for donors and partners to improve health system governance and leadership mechanisms in Afghanistan at the national and subnational level?	Delivery	0.86	0.76	0.76	0.87	0.71	**79.42%**	**0.78**

***Bold indicates that questions were not included in the top twenty ranked questions among the overall group.**

Table 7. Mapping of research priorities from CHRNI against the Health Sector Transition Strategy (2023-2025) strategic directions

Strategic Directions	Aligned Research Questions
**1 -** Strengthen and expand essential service coverage/utilization and quality of care and improve financial risk protection for the most vulnerable groups	Key activities include: reviewing the content of the BPHS/EPHS with a focus on improving efficiency and quality of care (aligned with research questions (RQs) 5 and 13); a service package review to understand the feasibility of integrating responses to public health emergencies such as COVID-19 and polio (RQ 1); expanding coverage of services to "white areas" through community-based strategies (RQs 3 and 8); developing an approach to financing community-based interventions, ensuring a package that would renumerate, motivate and recognize CHW work (RQs 3 and 10); and quality improvement of tertiary care (RQ 9), among others.

**2 -** Sustain and strengthen essential foundations of the health system that are necessary for meeting basic human health needs	Focus on strengthening four health systems building blocks: the health workforce, governance and coordination, health information systems, and supply chain management. In line with this were RQ 12 on practical strategies to strengthen human resources for health to improve service delivery and quality; RQ 20 on developing recommendations for donors and partners to improve health system governance and leadership mechanisms; and RQ 7 on challenges and solutions for consistent and accurate data collection and reporting within the health management information system.
**3 -** Strengthen the capacity to prevent, detect, and respond to disease outbreaks and other health emergencies	Focus on improving Afghanistan's health system resilience, preparedness, and capacity for emergency response, particularly in expanding and integrating disease surveillance systems. RQ 16, focusing on effective and sustainable strategies for communicable disease surveillance to support timely investigation, diagnosis, response, and prevention in Afghanistan, can help to bolster many of the outlined surveillance-related activities.
**4 -** Strengthen harmonization and alignment of financing toward national health priorities to increase predictability, adaptability, and efficiency of funding	Focus on strengthening the harmonization, coordination, and alignment of financing among government, international, humanitarian and development partners. Activities include establishing resource mapping at national and provincial levels, supporting national coordination of development and humanitarian partner investments, and supporting service delivery alignment at provincial level. RQ 14 focused on strategies for the effective and efficient management of humanitarian financial assistance to optimize health and health system outcomes, including in remote areas, would serve to inform these activities.

Figure 1. Primary Health Care Performance Initiative's Conceptual Framework

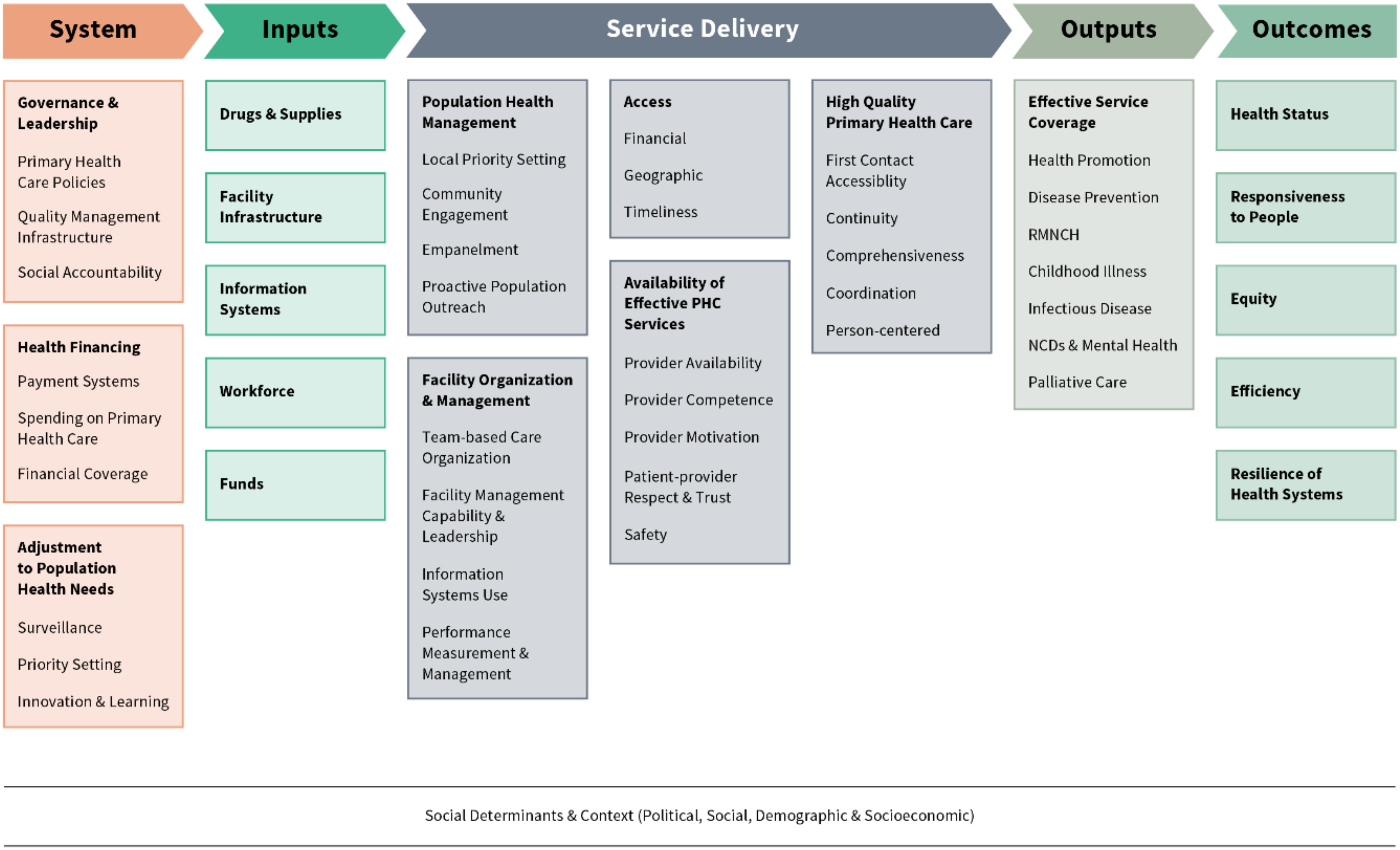

Supplement: online supplemental file 1 [file bmjgh-10-Suppl_3-s001.pdf]
